# Justus von Liebig: His Life and Work (1803-1873)

**Published:** 1899-06

**Authors:** 


**Justus von Liebig: his Life and Work** (1803–1873). By W. A. Shenstone. Pp. 219. London: Cassell and Company, Limited. 1895.

Mr. Shenstone deals in popular style with the life-work of one who made no less important contributions to science as a means of education than to the advancement of his special province—chemistry. Liebig's name will be associated in most minds with the relations of the chemical composition of the soil to the growth of plants; but his establishment of a teaching laboratory, and the dissemination of the results of the researches of master and pupils, have probably had an even more important influence on modern scientific progress.

This biography, which is issued in "The Century Science Series," will be read with the greatest interest by all who are concerned with the foundations of our present knowledge of chemistry in its relation to life, and Mr. Shenstone is to be congratulated on the success with which he has depicted the main scenes in a memorable career.

